# Efficacy of povidone-iodine nasal rinse and mouth wash in COVID-19 management: a prospective, randomized pilot clinical trial (*povidone-iodine in COVID-19 management*)

**DOI:** 10.1186/s12879-024-09137-y

**Published:** 2024-03-01

**Authors:** Saad Alsaleh, Ahmed Alhussien, Abduljabbar Alyamani, Fahad Alhussain, Ali Alhijji, Khalifa Binkhamis, Anas Khan, Amin Javer, Fatimah S. Alshahrani

**Affiliations:** 1 Otolaryngology - Head & Neck Surgery Department, College of Medicine, King Saud University, Riyadh, Saudi Arabia; 2 Department of Otolaryngology - Head and Neck Surgery, King Faisal Specialist Hospital and Research Center, Riyadh, Saudi Arabia; 3 Department of Urology, King Faisal Specialist Hospital and Research center, Riyadh, Saudi Arabia; 4 Division of Infectious Diseases, Department of Internal Medicine, College of Medicine, King Saud University and King Saud University Medical City, Riyadh, Saudi Arabia; 5 Department of Pathology, College of Medicine, King Saud University, Riyadh, Saudi Arabia; 6 Department of Emergency Medicine, College of Medicine, King Saud University, Riyadh, Saudi Arabia; 7 Division of Otolaryngology– Head and Neck Surgery, Faculty of Medicine, University of British Columbia, Vancouver, Canada

**Keywords:** Povidone-iodine, Saline solution, Nasal lavage, Mouth washing, COVID-19, SARS-CoV-2

## Abstract

**Objectives/Hypothesis:**

To assess the efficacy of 0.23% povidone-iodine (PVP-I) nasal rinses and mouth washes on detectability of the coronavirus disease 2019 (COVID-19) virus and cycle threshold (Ct) values in nasopharyngeal swabs.

**Study design:**

This was an open-label, prospective, randomized, placebo-controlled clinical trial.

**Setting:**

The study was conducted in King Saud University Medical City, Riyadh, Saudi Arabia, from August 2021 to July 2022.

**Methods:**

Participants diagnosed with SARS-CoV-2 were randomly assigned to one of three groups, with participants receiving either 0.23% PVP-I, 0.9% normal saline (NS) nasal rinses and mouth washes, or no intervention (control group). Nasopharyngeal swabs were taken 4, 8, 12, and 18 days after the first swab to measure the detectability of the virus and the Ct.

**Results:**

A total of 19 participants were involved in this study. The mean viral survival was 9.8, 12, and 12.6 days for the PVP-I, NS, and control groups, respectively, with a statistically significant difference (*p* = 0.046). The Ct mean values were 23 ± 3.4, 23.5 ± 6.3, and 26.3 ± 5.9 at the time of recruitment and 25.2 ± 3.5, 15 ± 11.7, and 26.9 ± 6.4 after 4 days for the PVP-I, NS, and control groups, respectively.

**Conclusions:**

When used continuously at a concentration of 0.23%, PVP-I showed promising results in terms of decreasing the pandemic burden by reducing the period of infectiousness and viral load. However, the use of PVP-I did not result in significantly different changes in the quality-of-life parameters in recently vaccinated and mild COVID-19 patients.

**Supplementary Information:**

The online version contains supplementary material available at 10.1186/s12879-024-09137-y.

## Introduction

At the end of 2019, the world began experiencing a new disease outbreak caused by a novel coronavirus that initially caused an epidemic in China. In March 2020, the World Health Organization (WHO) declared the disease a world pandemic and designated it as coronavirus disease 2019 (COVID-19), with the causative agent being severe acute respiratory syndrome coronavirus 2 (SARS-CoV-2) [1]. The COVID-19 pandemic is ongoing and, according to recent estimates, the number of cases exceeds 617 million, and there have been six million deaths due to COVID-19 [2].

The virus infects cells via angiotensin-converting enzyme 2, which is expressed weakly over the nasopharyngeal and oral cavity epithelium and most frequently found in type II alveolar cells of the lung, making epithelial lung cells the primary viral target [3, 4]. However, active viral replication occurs in the tissues of the upper respiratory tract as well, and pharyngeal shedding was found to be high during the first week of symptoms [5]. In addition, the nasopharynx was found to have a higher viral load than the oropharynx in affected patients, indicating that transmission can occur via both saliva and nasal secretions [6]. Therefore, the upper respiratory tract might be a key target for treatment and efforts to attenuate disease progression into the lower airway, reduce the severity of the disease, and decrease transmission.

Povidone-iodine (PVP-I) is a complex of polyvinylpyrrolidine and tri-iodine ions that is widely used as an antimicrobial agent on skin, mucous membranes, and wounds [7]. Topical application in the nose has been demonstrated to be clinically safe, tolerable, and effective against different types of bacteria, viruses, yeast, and protozoa [8].

Therefore, in this study, we aimed to assess the efficacy of 0.23% PVP-I nasal rinses and mouth washes in decreasing the viral load and duration of detectability of SARS-CoV-2 in nasopharyngeal swabs. We also aimed to determine whether there was an improvement in quality of life associated with using PVP-I by using two different tools: the Wisconsin Upper Respiratory Symptom Survey (WURSS)–11 and the Sino-Nasal Outcome Test-22 (SNOT-22) [9, 10].

## Materials and methods

### Study design and settings

The study was a prospective, randomized, open-label, placebo-controlled clinical trial with a 1:1:1 allocation ratio. It was conducted in King Saud University Medical City, Riyadh, Saudi Arabia, from August 2021 to July 2022.

### Participants

The recruited participants consisted of patients who were diagnosed with SARS-CoV-2 via real-time reverse-transcription polymerase chain reaction (RT-PCR) testing of nasopharyngeal swab samples according to clinical laboratory protocols and internal policies and who developed COVID-19-compatible symptoms within the past 72 h. We included patients who were recovering in their home and did not require hospitalization. We excluded patients who reported hypersensitivity to iodine or betadine, a history of any thyroid disorder (hyperthyroidism, hypothyroidism, thyroid nodule or cancer, or use of any thyroid medication, such as thyroxine or carbimazole), sinonasal disease requiring regular use of nasal rinses, sinonasal tumors, or use of topical iodine or betadine for any other reason. We also excluded patients who were pregnant or breastfeeding, hospitalized, younger than 18 years, elderly (more than 60 years old), or immunocompromised.

### Grouping and randomization

Participants were assigned to one of three groups. Participants in the first group underwent nasal rinses and mouth washes with 0.23% PVP-I twice daily for two weeks (PVP-I group), those in the second group underwent nasal rinses and mouth washes with 0.9% normal saline (NS) twice daily for two weeks (NS group), and those in the third group did not receive any intervention (control group). At the time of recruitment, the participants were randomly assigned a number from 1 to 36 using a random number generator website (http://random.org), and those numbered 1–12, 13–24, and 25–36 were placed in the PVP-I, NS, and control groups, respectively.

### Intervention

Nasal rinses were performed as irrigations by administering 60 mL of solution on each side using a syringe. Mouth washes were performed with 20 mL of solution for 10 s twice daily, with the PVP-I group using 0.23% PVP-I and the NS group using NS. A demonstrative video was provided to each participant in the first and second arm to ensure proper use of the rinses.

### Outcome measures

Nasopharyngeal swabs were performed by a nurse 4, 8, 12, and 18 days after the first swab, and the samples were subjected to RT-PCR to measure the detectability of the virus and the Ct. The resultant RT-PCR Ct values represent the number of amplification cycles required for the target gene to exceed a threshold level. Ct values are therefore inversely related to viral load and measuring Ct can be used as an indirect method for quantifying viral RNA copy number in a sample [11]. Additionally, subjective changes to the symptoms were measured using validated Arabic questionnaires: the WURSS-11 and the SNOT-22 [9, 10]. Throughout the intervention, we monitored participants’ compliance and tracked the progression of COVID-19 symptoms on a daily basis. We also assessed the need for hospitalization. Additionally, we closely monitored for any potential side effects, including discomfort, hypersensitivity, and symptoms related to hypothyroidism.

### Informed consent and ethical approval

All patients who met the inclusion criteria signed a written informed consent form that mentioned possible risks and benefits associated with the intervention. All procedures conducted in this study were in accordance with the ethical standards of the institutional and national research committees and with the 1964 Declaration of Helsinki. Approval was obtained from the King Saud University College of Medicine Institutional Review Board (number: E-20-5656) and the Saudi Food and Drug Authority (SFDA) Clinical Trials Unit (number: 21,060,203) before starting the study. The study was registered on clinicaltrials.gov with registration number NCT04449965 on 29/06/2020.

### Sample size and statistical analysis

In the design phase of this trial, reports of RT-PCR detectability and Ct values of SARS-CoV-2 were lacking in published literature. Therefore, the pilot trial was designed based on feasibility, precision (in terms of mean and variance), and regulatory considerations. We planned to include 12 patients in each group (12 = 10 + 2, including 20% compensation for dropouts) [12].

The mean and median duration of SARS-CoV-2 infectivity and the standard errors and 95% confidence intervals (CIs) were determined. The survival duration of the groups was compared using a log-rank (Mantel–Cox) test. Ct values were compared between all pairs using a nonparametric test (Wilcoxon signed-rank test). For the quality-of-life scores obtained using the WURSS-11 and SNOT-22, the change from the baseline SNOT-22 score was determined at every post-baseline visit, and the data were analyzed using repeated measure analysis of covariance (RMANCOVA). Lastly, *p*-values < 0.05 were considered statistically significant. SPSS 23 software was used for the statistical analysis.

## Results

### Demographics

**Fig. 1:**
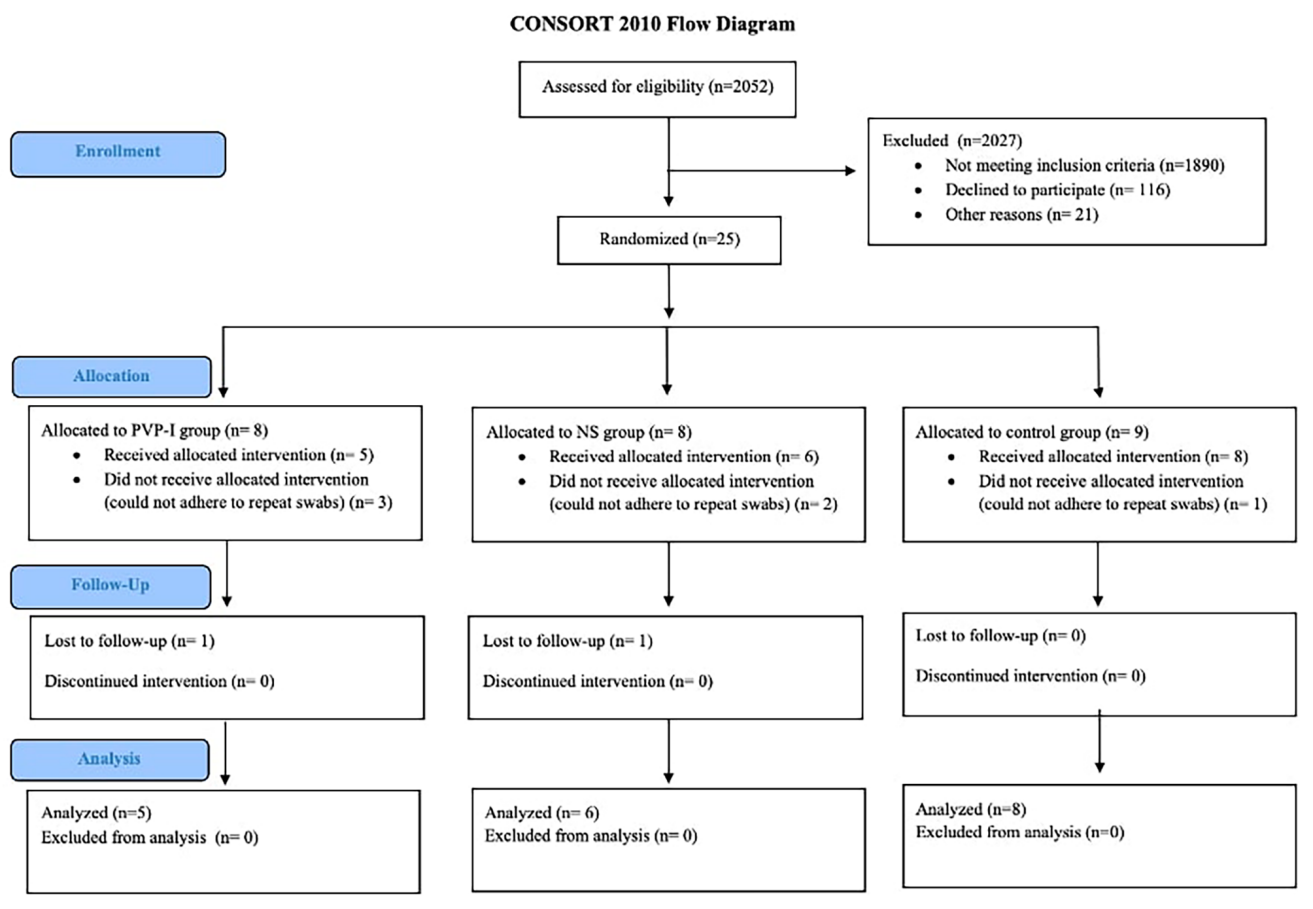
CONSORT flow diagram showing the randomized trial process [13]

Among the 2,052 patients who were newly diagnosed with COVID-19 and assessed for eligibility, only 25 patients were included and randomized in this trial. Of these 25, 19 participants were allocated to the three groups: five to the PVP-I group, six to the NS group, and eight to the control group. Two participants dropped out during follow-up; however, their data were included in the final analysis (Fig. 1).

There were 12 males and 7 females among the 19 participants. The age of the participants ranged from 18 to 42 years, and the median age was 38 years. All participants were vaccinated against COVID-19, and all except one participant had received more than one dose. Only 26.3% of the participants had a normal body mass index, 57.9% were overweight, and 15.8% were obese (Table 1).

**Table 1:** The participants’ demographic data

		PVP (*n* = 5)	NS (*n* = 6)	Control (*n* = 8)	Total (*n* = 19)
**Sex**	Male	3	4	5	12
	Female	2	2	3	7
**Age**	> 40 years	0	1	0	1
	30–39 years	4	1	5	10
	< 30 years	1	4	3	8
**Received vaccination**	1 dose	0	0	1	1
	2 doses	3	6	7	16
	3 doses	2	0	0	2
**Time since last vaccination**	< 3 months	1	2	4	7
	3–6 months	4	3	2	9
	> 6 months	0	1	2	3
**Body mass index**	18–25 kg/m^2^	1	1	2	4
	25–30 kg/m^2^	2	4	5	11
	30–35 kg/m^2^	0	0	1	1
	> 35 kg/m^2^	2	0	0	2

### Viral survival and viral load

**Fig. 2:**
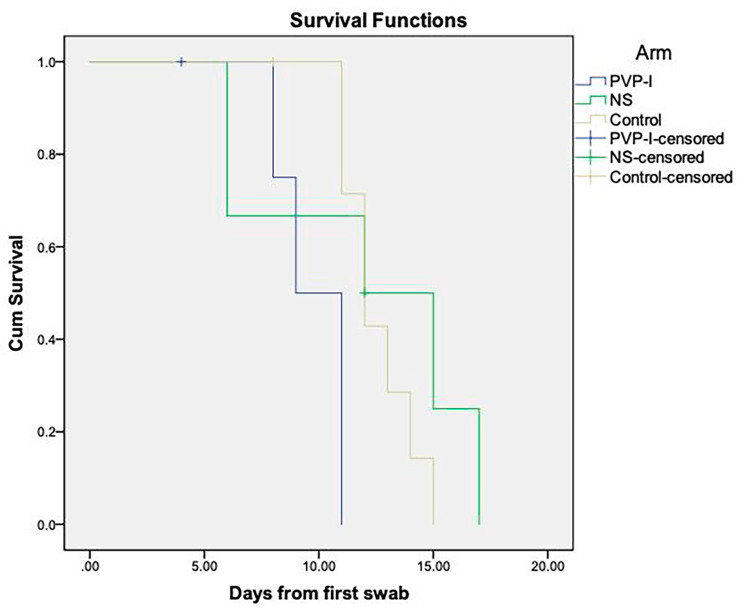
Survival curve plots showing days of viral survival in the PVP-I group (blue), NS group (green), and control group (yellow)

As shown in Fig. 2; Table 2, the mean viral survival was 9.8, 12, and 12.6 days for the PVP-I, NS, and control groups, respectively, and the difference was found to be statistically significant (*p =* 0.046). The Ct mean values were 23 ± 3.4, 23.5 ± 6.3, and 26.3 ± 5.9 at the time of recruitment and 25.2 ± 3.5, 15 ± 11.7, and 26.9 ± 6.4 after four days for the PVP-I, NS, and control groups, respectively. The differences between the Ct values were not statistically significant across the three groups, with *p*-values of 1, 0.07, and 0.83, respectively (Table 3).

**Table 2:** Survival analysis results derived using the Kaplan–Meier method. The number of viral survival days for each group is shown

	Days		p*
	Mean (SE) [CI]	Median (SE) [CI]	
**PVP-I group (n = 5)**	9.8 (0.8) [8.3–11.2]	9 (1) [7–11]	
**NS group (n = 6)**	12 (2.1) [7.9–16.1]	12 (4.4) [3.4–20.6]	0.046
**Control group (n = 8)**	12.6 (0.6) [11.5–13.7]	12 (0.7) [10.7–13.3]	

SE: Standard error; CI: Confidence interval; PVP-I: Povidone-Iodine; NS: Normal Saline

* Using log-rank (Mantel–Cox) test

* *p* < 0.05 considered statistically significant

**Table 3:** Cycle threshold (Ct) values recorded from the swab samples taken before intervention and four days after intervention. The analysis was performed using the Wilcoxon signed-rank test

	Mean Ct at Day 0 (SD)	Mean Ct at Day 4 (SD)	p*
**PVP-I group (n = 5)**	23 (± 3.4)	25.2 (± 3.5)	1.00
**NS group (n = 6)**	23.5 (± 6.3)	15 (± 11.7)	0.07
**Control group (n = 8)**	26.3 (± 5.9)	26.9 (± 6.4)	0.83
**Overall (n = 19)**	24.5 (± 5.4)	22.7 (± 9.3)	0.46

PVP-I: Povidone-Iodine; NS: Normal Saline

* *p* < 0.05 considered statistically significant

### Symptom changes

**Fig. 3:**
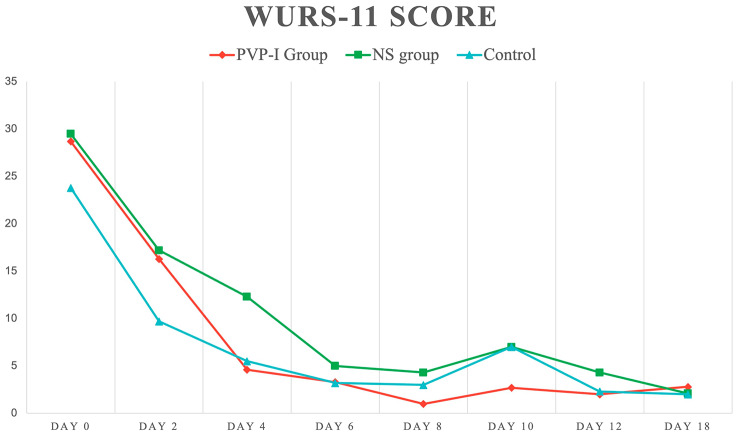
Line graph showing the Wisconsin Upper Respiratory Symptom Survey (WURSS)-11 mean score (Y-axis) and number of days since the first swab (X-axis)

The WURSS-11 scores dropped in the first four days from 28.7 ± 14 and 23.8 ± 12 to 4.6 ± 4 and 5.5 ± 5 in the PVP-I and control groups, respectively. However, the NS group required six days to reach a score of 5 ± 5. The difference in the scores of the three groups was not statistically significant (*p =* 0.75; Table 4; Fig. 3).

**Fig. 4:**
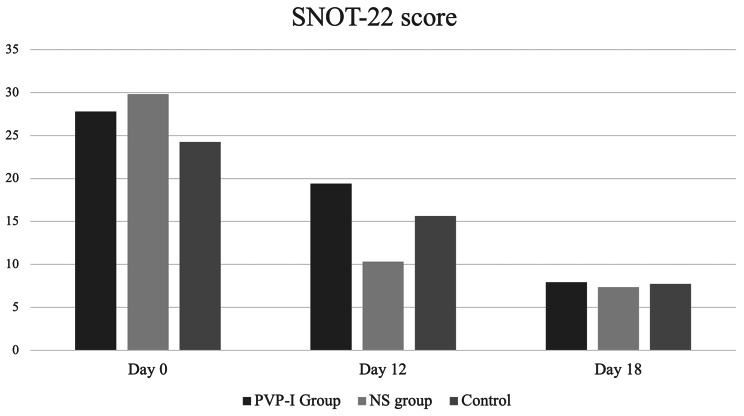
A bar graph illustrating the differences between the mean Sino-Nasal Outcome Test (SNOT)-22 scores for each group (Y-axis) at each time point (Days 0, 12, and 18)

Improvement in the SNOT-22 scores of the three groups required a longer duration of up to 18 days. When the changes in the scores were compared, no statistically significant difference was found between the PVP-I, NS, and control groups (*p* = 0.08; Table 5; Fig. 4).

**Table 4:** The Wisconsin Upper Respiratory Symptom Survey (WURSS)-11 scores recorded for each group at different times. The score reflects the daily symptoms

	Day 0	Day 2	Day 4	Day 6	Day 8	Day 10	Day 12	Day 18	p*
**PVP-I group**	28.7 ± 14	16.3 ± 6	4.6 ± 4	3.3 ± 3	1 ± 1	2.7 ± 3	2 ± 1	2.8 ± 4	
**NS group**	29.5 ± 19	17.2 ± 11	12.3 ± 6	5 ± 5	4.3 ± 4	7 ± 7	4.3 ± 4	2.1 ± 3	0.75
**Control group**	23.8 ± 12	9.7 ± 5	5.5 ± 5	3.2 ± 3	3 ± 4	7 ± 7.1	2.3 ± 4	2 ± 3.5	
**Total**	27.1 ± 15	14 ± 9	8.1 ± 6	3.9 ± 4	3.1 ± 3	4 ± 5	3.1 ± 4	2.2 ± 3	< 0.001

PVP-I: Povidone-Iodine; NS: Normal Saline

* Using repeated measures ANOVA

* *p* < 0.05 considered statistically significant

**Table 5:** The Sino-Nasal Outcome Test (SNOT)-22 scores recorded for each group at different times

	Day 0	Day 12	Day 18	p*
**PVP-I group**	27.8 ± 25.3	19.4 ± 21	7.9 ± 13.9	
**NS group**	29.8 ± 11.7	10.3 ± 7.7	7.3 ± 7.8	0.08
**Control group**	24.3 ± 14.3	15.6 ± 19.1	7.7 ± 10.9	
**Total**	26.9 ± 14	15 ± 14.5	7.7 ± 8.2	< 0.001

PVP-I: Povidone-Iodine; NS: Normal Saline

* *p* < 0.05 considered statistically significant

## Discussion

The COVID-19 pandemic has changed the environment in which medicine is practiced. Efforts to minimize the viral spread have concentrated on implementing physical barriers. However, the oropharynx and nasopharynx have a high viral load, and decontamination of these areas could play an important role in improving the disease condition and decreasing transmission of the virus. The value of PVP-I in the inactivation of SARS-CoV-2 was previously proven in vitro. Two different studies indicated that a 0.5% solution was sufficient for virus inactivation [14, 15]. Frank et al. proved that contact for as little as 15 s is effective. No cytotoxic effects were observed at any concentration or duration of contact in either study [16, 17]. However, we selected the lowest effective concentration of PVP-I studied in vitro against SARS-CoV to establish the safest and most tolerable concentration for use in the PVP-I nasal route [18].

In our study, we found a significantly higher rate of SARS-CoV-2 clearance in the PVP-I group, represented by lower detectability duration in nasopharyngeal swab samples, compared to the NS and control groups. To our knowledge, the concentration of PVP-I used in this study (0.23%) is lower than that used in any other published studies against SARS-CoV-2, and our findings are consistent with those of several other clinical trials [19–25]. In addition, we studied the long-term and continuous effect of PVP-I (up to 18 days) on virus survival. We observed that continuous exposure to PVP-I—even at low concentrations—can decrease the duration of virus detectability and viral load more than exposure to NS and no intervention. These findings align with those of a meta-analysis that studied different types of nasal rinses and mouth washes used against SARS-CoV-2 and included 22 in vitro studies and 11 in vivo studies. Among the in vivo studies, PVP-I was the most studied solution and was found to be the most effective regardless of concentration, with a Log_10_ reduction value of nearly 3. The results of the in vivo studies were consistent with those of the in vitro studies, with PVP-I displaying the highest mean reduction in viral load (0.86) among all the tested solutions [14].

Ct values determined using RT-PCR represent the number of amplification cycles required for a target gene to exceed a threshold level. Ct values are therefore inversely related to viral load, and measuring Ct values can be an indirect method for quantifying viral RNA copy number in a sample. A systematic review by Rao et al. concluded that a low Ct may be a useful tool for predicting disease severity and prognosis in patients with COVID-19 [26]. In our study, the use of PVP-I resulted in an increase in Ct values, which suggests that it contributed to a decline in the disease severity and an improvement in the prognosis. Also, a minor increase of Ct values was observed in control group. The use of NS resulted in a decrease in Ct values. However, these changes were not statistically significant.

The efficacy of COVID-19 vaccines is continuously under threat because new variants of the causative agent, SARS-CoV-2, are constantly arising. Additionally, the influenza virus exhibits antigenic drift and shift, which means that there is a constant need to update the composition of the inactivated influenza vaccines [27]. Therefore, there is also a need to develop effective and accessible treatments for a range of viruses that threaten human health, such as nasal rinses and mouth washes. PVP-I was tested in an in vitro study against severe acute respiratory syndrome-associated coronavirus (SARS-CoV), Middle East respiratory syndrome coronavirus (MERS-CoV), rotavirus strain Wa, and influenza virus A subtype H1N1, and it was found that PVP-I at a concentration of 0.23% rapidly inactivated all the viruses after 15 s of exposure [28]. These data support further study and use of topical PVP-I against different types of upper respiratory tract viruses that share similar pathophysiology with COVID-19.

PVP-I is considered to be safe for use in the nose and on the oral mucosa. Frank et al. concluded in their review that PVP-I can be safely used at concentrations of up to 1.25% and 2.5% for the oral and nasal cavities, respectively [18]. In a study of 12 patients who used mouth washes containing 5% PVP-I, it was found that all the patients had an increase in the level of thyroid stimulating hormone (TSH), with five having levels that exceeded the normal limit. However, no alterations in triiodothyronine or thyroxine were noted [29]. In another study, conducted by Guenezan et al., patients who used solutions containing 0.5% PVP-I reported local side effects described as nasal tingling [30]. In our study, no patient reported any nasal discomfort after PVP-I usage. This might be explained by the low concentration of 0.23%. TSH levels were not measured before and after intervention. However, no patients reported any symptoms of hypothyroidism.

Arakeri et al. suggested that 0.5% PVP-I showed an inhibitory effect on leukotriene B4 and leukocyte extravasation (chemotaxis), which resulted in an anti-edematous effect in a clinical trial conducted after tooth extraction [31]. In contrast, two other clinical trials found no significant differences in the SNOT-22 scores of patients with chronic rhinosinusitis after endoscopic nasal surgery who used or did not use 0.1% PVP-I [32, 33]. Additionally, Zarabanda et al. found a more favorable course of improvement in UPSIT scores and olfaction among COVID-19 patients who used PVP-I nasal rinses (0.5% and 2%) compared to the placebo group. However, their findings did not result in statistical significance between the groups [25]. These findings are similar to ours, as we did not find a significant difference between the PVP-I and control groups in terms of symptom severity and quality of life. This might be due to the lower dose of PVP-I used in our study compared to that used by Arakeri et al. Another factor that could explain this is that participants included in this study had received at least one vaccine dose. As has been proven in multiple trials, different types of vaccines significantly decrease the severity of COVID-19 symptoms [34].

Although the findings of this study are encouraging, several limitations should be noted. First, RT-PCR and Ct values were used as markers for viral survival and viral load, and these have limitations in their capacity to detect virus viability compared to viral culture [35]. Second, we faced recruitment difficulties. These included but were not limited to the emergence of COVID-19 home tests, which decrease the detectability of infected people. Also, recently, people have lost interest in being diagnosed and receiving any intervention for COVID-19 [36]. Third, as the pattern of COVID-19 infection occurs as waves, the availability of laboratory facilities for research is compromised when cases are high. Therefore, more studies with larger sample sizes are warranted, as is the use of viral culture methods and standardization of the timing and the sample population.

## Conclusions

Within the limitations of the current study, PVP-I is a cheap, widely available, and safe topical medication that has shown promising results when used continuously at a concentration of 0.23% to decrease the pandemic burden by reducing the period of infectiousness and viral load. Also, it might be a potential alternative to home isolation which can result in a significant reduction in economic and social burdens. However, the use of PVP-I did not result in statistically significant differences in sinonasal symptoms and quality-of-life parameters in recently-vaccinated and mild COVID-19 patients.

### Electronic supplementary material

Below is the link to the electronic supplementary material.

Supplementary Material 1

## Data Availability

All data generated and analysed during this study are included in this published article and its supplementary information files.
